# The lack of public health research output from India

**DOI:** 10.1186/1471-2458-4-55

**Published:** 2004-11-25

**Authors:** Lalit Dandona, Yegnanarayana S Sivan, Mukkamala N Jyothi, VS Udaya Bhaskar, Rakhi Dandona

**Affiliations:** 1 Centre for Public Health Research, Administrative Staff College of India, Raj Bhavan Road, Hyderabad – 500 082, India

## Abstract

**Background:**

Systematic assessment of recent health research output from India, and its relation with the estimated disease burden, is not available. This information would help understand the areas in health research that need improvement in India to enhance the health of India's population.

**Methods:**

The health research output from India during 2002, which was accessible in the public domain, was assessed by searching PubMed and other internet health literature databases, and was related to the disease burden suggested by the Global Burden of Disease Study. The main outcome measures were number of health papers with abstracts in basic, clinical and public health sciences; quality-adjusted research output based on the impact factors of journals in which the papers were published; classification of papers in disease/condition categories and comparison of research output with the estimated disease burden in each category. Comparison of the health papers from India during 2002 included in PubMed was done with those from Australia during one quarter of 2002.

**Results:**

Of the 4876 health papers from India in 2002 in PubMed, 48.4%, 47.1% and 4.4% were in basic, clinical and public health sciences, respectively. Of the 4495 papers based on original research, only 3.3% were in public health. Quality-adjusted original research output was highest for non-communicable diseases (62% of total). Of the total quality-adjusted original research output, the proportions in injuries (0.7%), cardiovascular diseases (3.6%), respiratory infections (0.2%), diarrhoeal diseases (1.9%), perinatal conditions (0.4%), childhood cluster diseases (0.5%), unipolar major depression (0%), and HIV/AIDS (1.5%) were substantially lower than their proportional contribution to the disease burden in India. Human resources, health policy, health economics, and impact assessment of interventions were particularly poorly represented in public health research. The Australia-India ratio for quality-adjusted health research output per unit gross domestic product was 20 and for public health research output was 31.

**Conclusions:**

Good-quality public health research output from India is grossly inadequate, and strategic planning to improve it is necessary if substantial enhancement of population health were to be made possible. There is inordinately low relative research output in several diseases/conditions that cause major disease burden in India.

## Background

India suffers a large proportion of the disease burden of the world, which has been estimated to be more than its 16.8% share of the world's population [1,2]. One of the vital elements in improving this situation is the need for a comprehensive and relevant evidence base that would equip India to take informed actions. A systematic assessment of recent health research output from India is not available. Without objective information about the current deficiencies and strengths in the health research output from India, it is difficult to plan substantial improvements in health research output that could enhance India's health status. We analysed the health research output from India in 2002 and related it with the estimated disease burden to identify areas that require particular attention to facilitate effective action to reduce disease burden in this world's second most populous country.

## Methods

Health research output was defined as tangible research information related to human health that was readily accessible in the public domain. PubMed [3,4] (which includes MEDLINE) of the US National Library of Medicine, the most widely used online health literature search database in the world, and websites of major academic institutions in India, international agencies, and publishing houses, were searched to ascertain the health research output from India in the year 2002.

PubMed was searched for papers published from India in 2002 using "India" in the author *affiliation *option in PubMed for all journals, and also by searching the Indian journals in PubMed as several papers in these journals mention only city and state but not India in the author affiliation. Only papers with abstracts were included, as the aim was to review all abstracts and classify the papers in various categories, including type of research, type of paper, disease/condition covered, allopathic or traditional system of medicine, and type and location of first author's institution. PubMed gives institutional affiliation and its location only for the first author. Papers that showed the first author affiliation with an Indian institution were considered as research output from India.

Definitions were used to classify the Indian papers located in PubMed. Health research was defined as research that could be related to health. *Basic research *was considered either *pure *or *applied*, pure being experimental or theoretical work to advance health knowledge without a defined specific application and applied having such an application. *Clinical research *was categorised as *patient series*/*management *if the paper was about clinical cases or issues in management of patients, *laboratory *if it dealt mainly with laboratory analysis of patient specimens, *clinical trial *if it was a trial in the clinical setting, and *clinical epidemiology *if it was about distribution and determinants of disease assessed in the clinical setting. *Public health research *was categorised into *epidemiology*, *environment/ social*, and *health systems*/*policy*. Epidemiology included *population epidemiology *that dealt with study of distribution and determinants of disease and health in the population, and *biostatistics*/*methods *that dealt with methodological issues in epidemiology. Environment/social included *environmental sciences *that dealt with environmental influences on health, and *social aspects *that dealt with social dimensions of health. Health system/policy included *health services *that dealt with aspects of health service provision, and *health policy *that dealt with concepts and frameworks related to the health system. A paper was classified as *original research *if it had original data collection and its analysis, and *review*/*viewpoint *if it was not based on original data. An attempt was made to classify each paper under the disease/condition that it covered, according to the listing used in the Global Burden of Disease Study [2]. If a paper covered generic issue(s) which could not be classified under a particular disease/condition, it was considered unclassifiable for disease/condition.

The 2002 impact factor of the journal, in which each paper was published, was used as a measure of the quality of each paper [5,6]. The proportion of papers and the quality-adjusted output for the diseases/conditions were related to the proportion of burden caused by each disease/condition in India as estimated for 2000 by the Global Burden of Disease Study [2]. The publications of 2002 were related to the disease burden of 2000, as research initiation to publication may take on an average a couple of years.

Percent quality-adjusted research output was calculated for papers in the categories of several classifications as follows:



IndMED [7], an online database of the Indian Medlars Centre, which covers several Indian biomedical journals was also searched. However, this database could not be included in the study, as the abstracts/papers for all the months of 2002 were not included in this database with substantial portions missing.

As reports on commissioned research in public health may be available on the websites of agencies/organisations, the websites of several international agencies (DFID, European Commission, UNAIDS, USAID, WHO, World Bank), twelve academic institutions of India involved with public health, and sixteen publishing houses, were searched to locate research on public health reported in the public domain from India in 2002.

For comparison of the Indian health research output with a developed country, a PubMed search was also done for papers published from Australia during the April–June 2002 quarter, using "Australia" OR "names/abbreviations of the states and territories of Australia [8]" in the author *affiliation *option in PubMed for all journals as several papers in Australian journals mentioned only city and state but not Australia in the author affiliation.

Data were entered in an MS Access database and analysed using SPSS software.

## Results

Of the 5718 papers with abstracts located on PubMed that were published from India in 2002, 842 (14.7%) papers were considered as non-health papers as they were on pure botany, chemistry, physics or zoology that could not be related to human health, and the other 4876 were health papers. The distribution of the types of research and the types of papers for the health papers is shown in Table 1. The basic and clinical science papers predominated, with public health papers comprising a very small fraction (4.4% of the total). The proportion of papers based on original research was substantially lower for public health (68.5%) than for basic sciences (94.4%) and clinical sciences (92.3%); of the total 4495 original research papers, public health made up only 3.3%. 4700 (96.4%) of the total health papers were on the allopathic system of medicine and 176 (3.6%) on the traditional systems of medicine in which the majority were on ayurveda (144 [81.8%]).

**Table 1:** Distribution of the types of health research and papers from India in 2002 included in PubMed

**Type of research**	No. (%)* of papers	**Type of paper**	
		
		**Original research No. [%]† (%)‡**	**Review / Viewpoint No. [%]† (%)‡**
Basic science	2358 (48.4)	2227 [49.6] (94.4)	131 [34.3] (5.6)
Pure	525 (10.8)	518 [11.5] (98.7)	7 [1.8] (1.3)
Applied	1833 (37.6)	1709 [38.0] (93.2)	124 [32.5] (6.8)
Clinical science	2296 (47.1)	2119 [47.2] (92.3)	177 [46.3] (7.7)
Patient series / management	1805 (37.0)	1639 [36.5] (90.8)	166 [43.5] (9.2)
Laboratory	283 (5.8)	277 [6.2] (97.9)	6 [1.6] (2.1)
Clinical trials	155 (3.2)	153 [3.4] (98.7)	2 [0.5] (1.3)
Clinical epidemiology	53 (1.1)	50 [1.1] (94.3)	3 [0.8] (5.7)
Public health	216 (4.4)	148 [3.3] (68.5)	68 [17.8] (31.5)
Epidemiology	85 (1.7)	72 [1.6] (84.7)	13 [3.4] (15.3)
Social / environmental	38 (0.8)	31 [0.7] (81.6)	7 [1.8] (18.4)
Health systems / policy	93 (1.9)	45 [1.0] (48.4)	48 [12.6] (51.6)
Other§	6 (0.1)	0 [0.0] (0.0)	6 [1.6] (100.0)
**Total**	**4876 (100)**	**4494 [100.0] (92.2)**	**382 [100.0] (7.8)**

*Percent of the total 4876 papers

**†**Percent of total in each type of paper

**‡**Percent of total in each type of research

§Papers that could not be classified in the above categories of type of research; these mostly consisted of biographies of persons or organizations

Table 2 shows the distribution of the diseases/conditions covered by the original research papers from India as compared with the estimated disease burden. A large proportion of the basic science papers (49%) were not classifiable into specific disease/condition categories, as they were generic in nature, as compared with 2.9% papers in clinical science and 13% in public health. Overall, the relative proportion of quality-adjusted original research output for non-communicable diseases was higher than their relative contribution to the disease burden, and this was most marked for clinical sciences. However, some major categories/sub-categories within non-communicable diseases were not covered adequately, as a fairly large proportion of research output was on conditions or issues that were not contributing as much to the disease burden. For example, cardiovascular diseases with a disease burden of 11.4% of the total in 2000 had a relatively low quality-adjusted research output of 3.6% of the total. The estimated disease burden due to neuro-psychiatric conditions was 9.6% of the total and the quality adjusted original research output in this category was relatively fair at 8.8%, but the two major sub-categories of unipolar major depression and biopolar disorder that made up 5.2% of the total disease burden had only 0.2% of the total quality-adjusted original research output. A similar mismatch was seen for infectious & parasitic diseases and respiratory infections that had 33.3% of the total quality-adjusted original research output for 33.9% of the total disease burden, but the six major sub-categories under this group contributing 30.1% of the total disease burden had only 11.8% of the total quality-adjusted original research output (Table 2).

**Table 2:** Distribution of original research health papers from India as compared with the estimated disease burden

Disease / Condition*	% DALY loss in 2000*	% DALY loss in 2010*	**No. (%) of original research health papers†**	**% quality-adjusted output for original research health papers‡**	**No. (%) of original research basic science papers§**	**% quality-adjusted output for original research basic science papers¶**	**No. (%) of original research clinical science papers#**	% quality-adjusted output for original research clinical science papers**	**No. (%) of original research public health papers††**	**% quality-adjusted output for original research public health papers‡‡**
Communicable, Maternal, Perinatal and Nutritional Conditions	44.2	34.1	950 (28.6)	37.4	397 (34.9)	42.9	484 (23.5)	29.1	69 (53.9)	59.4
Infectious & parasitic diseases	25.9	22.7	762 (22.9)	33.1	358 (31.5)	40.2	362 (17.6)	23.6	42 (32.8)	48.6
Tuberculosis	6.8	7.0	143 (4.3)	7.4	49 (4.3)	7.2	87 (4.2)	5.6	7 (5.5)	29.2
STDs excluding HIV	1.5	1.1	13 (0.4)	0.3	1 (0.1)	0.1	12 (0.6)	0.6	0 (0.0)	0.0
HIV	3.3	6.0	48 (1.4)	1.6	14 (1.2)	1.5	29 (1.4)	1.8	5 (3.9)	1.2
Diarrhoeal diseases	6.7	4.2	34 (1.0)	1.9	17 (1.5)	2.2	16 (0.8)	1.8	1 (0.8)	0.3
Childhood cluster diseases	4.1	2.5	12 (0.4)	0.5	4 (0.4)	0.4	5 (0.2)	0.6	3 (2.3)	0.0
Respiratory infections	8.0	5.0	18 (0.5)	0.2	2 (0.2)	0.1	15 (0.7)	0.4	1 (0.8)	0.0
Lower respiratory infections	7.7	4.9	8 (0.2)	0.1	2 (0.2)	0.1	6 (0.3)	0.2	0 (0.0)	0.0
Maternal conditions	1.4	0.6	84 (2.5)	1.8	17 (1.5)	1.1	60 (2.9)	2.4	7 (5.5)	2.4
Perinatal conditions	6.1	3.9	25 (0.8)	0.4	1 (0.1)	0.1	23 (1.1)	0.8	1 (0.8)	1.0
Nutritional deficiencies	2.9	1.8	45 (1.4)	1.4	8 (0.7)	0.5	22 (1.1)	1.8	15 (11.7)	7.1
Protein energy malnutrition	1.2	0.7	6 (0.2)	0.2	1 (0.1)	0.0	1 (0.0)	0.0	4 (3.1)	3.4
Iron deficiency anaemia	1.5	1.0	10 (0.3)	0.2	1 (0.1)	0.3	6 (0.3)	0.2	3 (2.4)	0.0
Noncommunicable diseases	38.7	47.5	2344 (70.6)	62.0	732 (64.4)	56.5	1555 (75.6)	70.1	57 (44.5)	40.2
Malignant neoplasms	3.8	5.4	370 (11.1)	11.2	118 (10.4)	9.1	251 (12.2)	14.4	1 (0.8)	2.9
Diabetes mellitus	0.8	0.8	129 (3.9)	3.2	64 (5.6)	3.6	57 (2.8)	2.3	8 (6.3)	8.5
Neuro-psychiatric conditions	9.6	11.5	248 (7.5)	8.8	112 (9.9)	10.5	124 (6.0)	6.6	12 (9.4)	11.6
Unipolar major depression	4.0	5.0	0 (0.0)	0.0	0 (0.0)	0.0	0 (0.0)	0.0	0 (0.0)	0.0
Bipolar disorder	1.2	1.4	3 (0.1)	0.2	0 (0.0)	0.0	1 (0.0)	0.1	2 (1.6)	3.5
Sense organ diseases	1.5	2.1	185 (5.6)	4.8	25 (2.2)	2.1	148 (7.2)	7.5	12 (9.4)	8.8
Cataract	1.2	1.7	25 (0.8)	0.9	6 (0.5)	0.3	16 (0.8)	1.3	3 (2.3)	2.5
Cardiovascular diseases	11.4	14.6	203 (6.1)	3.6	38 (3.3)	2.3	159 (7.7)	5.2	6 (4.7)	0.7
Ischaemic heart disease	5.3	7.1	56 (1.7)	0.9	11 (1.0)	0.7	41 (2.0)	1.2	4 (3.1)	0.2
Cerebrovascular disease	2.1	2.7	20 (0.6)	0.3	3 (0.3)	0.2	17 (0.8)	0.4	0 (0.0)	0.0
Respiratory diseases	3.7	4.8	68 (2.0)	1.5	15 (1.3)	1.1	45 (2.2)	1.6	8 (6.3)	3.2
Chronic obstructive pulmonary disease	1.4	2.0	2 (0.1)	0.0	0 (0.0)	0.0	2 (0.1)	0.0	0 (0.0)	0.0
Digestive tract diseases	2.3	2.4	198 (6.0)	5.3	52 (4.6)	3.7	143 (6.9)	7.5	3 (2.3)	1.5
Cirrhosis of liver	1.1	1.2	12 (0.4)	0.6	1 (0.1)	0.6	11 (0.5)	0.6	0 (0.0)	0.0
Congenital anomalies	3.4	3.5	105 (3.2)	1.6	2 (0.2)	0.2	103 (5.0)	3.3	0 (0.0)	0.0
Injuries	17.2	18.4	28 (0.8)	0.7	7 (0.6)	0.6	19 (0.9)	0.8	2 (1.6)	0.4
Unintentional injuries	15.0	15.9	24 (0.7)	0.6	7 (0.6)	0.6	15 (0.7)	0.6	2 (1.6)	0.4
Road traffic injuries	3.5	5.1	2 (0.1)	0.0	0 (0.0)	0.0	2 (0.1)	0.0	0 (0.0)	0.0
Falls	3.5	3.0	0 (0.0)	0.0	0 (0.0)	0.0	0 (0.0)	0.0	0 (0.0)	0.0
Fires	2.1	2.0	4 (0.1)	0.1	2 (0.2)	0.1	2 (0.1)	0.2	0 (0.0)	0.0
Intentional injuries	2.1	2.5	1 (0.0)	0.1	0 (0.0)	0.0	1 (0.0)	0.1	0 (0.0)	0.0
Self-inflicted injuries	1.4	1.7	1 (0.0)	0.1	0 (0.0)	0.0	1 (0.0)	0.1	0 (0.0)	0.0
**Total**	**100**	**100**	**3322 (100)**	**100**	**1136 (100) **	**100**	**2058 (100) **	**100**	**128 (100)**	**100**

*According to the Global Burden of Disease Study [2]; only diseases/conditions with disease burden of >1% of the total are listed, plus diabetes mellitus; since all diseases/conditions are not listed, the sum of sub-categories shown may not add up to the total for their categories; DALY is disability-adjusted life year

**†**Denominator for this percent calculation is 3322, which excludes 1172 papers that were not classifiable into specific disease/condition categories

‡ Based on the total impact factor of 3456.262 for the 3322 original health research papers included in this table

§Denominator for this percent calculation is 1136, which excludes 1091 papers that were not classifiable into specific disease/condition categories

¶Based on the total impact factor of 1757.491 for the 1136 original basic health research papers included in this table

#Denominator for this percent calculation is 2058, which excludes 61 papers that were not classifiable into specific disease/condition categories

**Based on the total impact factor of 1557.811 for the 2058 original clinical health research papers included in this table

**††**Denominator for this percent calculation is 128, which excludes 20 papers that were not classifiable into specific disease/condition categories

‡‡Based on the total impact factor of 140.96 for the 128 original public health research papers included in this table

Overall, the diseases/conditions that were substantially underrepresented in the relative proportion of quality-adjusted original research output as compared with their contribution to the disease burden were injuries, cardiovascular disease, respiratory infections, diarrhoeal diseases, perinatal conditions, childhood cluster diseases (including measles and tetanus), unipolar major depression, and HIV/AIDS (Table 2).

**Figure 1:**
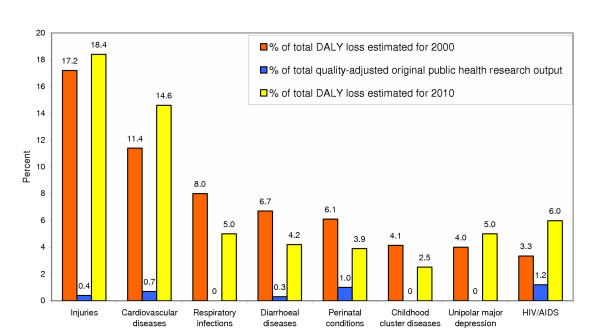
Diseases/conditions poorly represented in original public health research relative to their contribution to the disease burden in India.

As the research output was least in public health, a brief description follows to understand this deficiency better. Figure 1 shows the diseases/conditions that were estimated to contribute more than 4% of the total disease burden in 2000 or 2010, and for which the original research output in public health was less than one-third of their proportional contribution to the disease burden estimated for 2010, suggesting that these diseases/conditions needed particular attention. Table 3 shows the distribution of original research in the various areas of public health, which suggests that original research in human resources, health policy, and health economics is relatively more deficient within the already low public health research output. Only six of the original public health research papers were on assessing interventions across the various areas, suggesting that the existing public health research in India has not yet evolved to the stage of methodically assessing the impact of public health interventions, which is a necessary step in the evolution of effective public health action.

**Table 3:** Distribution of the types of original public health research from India

**Type of public health research**	**No. (%) of original research public health papers**	**Total impact factor of original research public health papers in each type**	**% quality-adjusted output for all original research public health papers†**
Epidemiology	72 (48.6)	59.6	38.9
Population epidemiology	69 (46.6)	56.5	36.9
Biostatistics / Methods	3 (2.0)	3.0	2.0
Environment / Social	31 (20.9)	29.3	19.1
Environmental sciences	14 (9.5)	16.7	10.9
Social aspects	17 (11.5)	12.7	8.3
Health Systems / Policy	45 (30.4)	64.4	42.0
Health services	42 (28.4)	63.3	41.3
Health economics*	8 (5.4)	5.7	3.7
Training / human resources*	5 (3.4)	0.6	0.4
Health policy	3 (2.0)	1.1	0.7
**Total**	**148 (100)**	**153.3**	**100**

*Health economics and training / human resources are sub-categories of health services

**†**Based on the denominator of 153.3

Of the total 4876 health papers from India in PubMed for 2002, 1300 (26.7%) were published in Indian journals, but these papers accounted for only 1.5% of the total impact factor of all health papers from India due to the very low impact factors of Indian journals. Among the public health papers 44.4% were published in Indian journals, for clinical sciences papers this was 39.7%, whereas this proportion was much smaller for basic sciences (12.4%).

The highest proportion of quality-adjusted basic research output was by university departments, institutions affiliated with the Council of Scientific and Industrial Research, and technical institutions; the predominant proportion of clinical research was by medical colleges / hospitals; and public health research by medical colleges / hospitals, government departments (due to one paper in a very high impact factor journal), and institutions affiliated with the Indian Council of Medical Research (Table 4). The National Capital Territory of Delhi accounted for the highest health research output among all states / union territories or cities (Table 5). The top ten research producing cities, with 6% of the population of India, produced 75.6% of the quality-adjusted research output, suggesting a concentration of quality research activity in parts of the country.

**Table 4:** Distribution of health research output from various types of institutions in India

**Type of institution**	**All health**		**Basic science**		**Clinical science**		**Public health**	
	
	**No. (%) of health papers**	**% quality-adjusted output for health papers**	**No. (%) of basic science papers**	**% quality-adjusted output for basic science papers**	**No. (%) of clinical science papers**	**% quality-adjusted output for clinical science papers**	**No. (%) of public health papers**	**% quality-adjusted output for public health papers**
Medical college / Hospital	2571 (52.7)	33.4	407 (17.3)	11.9	2044 (89.0)	82.1	119 (55.1)	45.9
Indian Council of Medical Research*	159 (3.3)	4.0	52 (2.2)	2.3	78 (3.4)	6.7	28 (13.0)	13.1
Council of Scientific and Industrial Research**†**	387 (7.9)	13.3	361 (15.3)	18.9	21 (0.9)	1.7	5 (2.3)	0.6
Technical institutions**‡**	281 (5.8)	12.2	268 (11.4)	17.7	7 (0.3)	0.6	5 (2.3)	2.3
Paramedical college/institution	164 (3.4)	2.9	154 (6.5)	4.1	8 (0.3)	0.4	2 (0.9)	0.9
University department	813 (16.7)	16.4	743 (31.5)	23.0	53 (2.3)	2.5	16 (7.4)	4.7
NGO / Foundation / Society	69 (1.4)	1.2	12 (0.5)	0.6	33 (1.4)	1.6	22 (10.2)	8.0
Government department	4 (0.1)	0.6	0 (0.0)	0.0	0 (0.0)	0.0	4 (1.9)	17.9**§**
Industry	31 (0.6)	0.6	28 (1.2)	0.9	3 (0.1)	0.1	0 (0.0)	0.0
Other	397 (8.1)	15.4	333 (14.1)	20.6	49 (2.1)	4.3	15 (5.9)	6.7
**Total**	4876 (100)	100	2358 (100)	100	2296 (100)	100	216 (100)	100

*Institutions affiliated with the Indian Council of Medical Research [15]

**†**Institutions affiliated with the Council of Scientific and Industrial Research [16]

**‡**Indian Institutes of Technology, Indian Institute of Science, and other technical institutions

**§**Percentage high due to one paper in a very high impact factor journal

The total of basic, clinical and public health papers does not add up to the "all health" papers in all rows, as 6 "other" papers that could not be classified as basic, clinical or public health (Table 1) are not included in this table

**Table 5:** Distribution of health research output from states and cities in India

State / Union Territory*	**Population in millions†**	**No. (%)‡ of health papers**	**No. of health papers per million population**	**Total impact factor of health papers**	**% quality-adjusted health research output§**	**Total impact factor per million population**
National Capital Territory of Delhi	13.8	1014 (20.8)	73.5	1216.0	20.8	88.1
Karnataka	52.7	491 (10.1)	9.3	786.8	13.5	14.9
Maharashtra	96.7	573 (11.8)	5.9	710.1	12.2	7.3
Uttar Pradesh	166.0	484 (9.9)	2.9	591.0	10.1	3.6
West Bengal	80.0	362 (7.4)	4.5	510.6	8.7	6.4
Tamil Nadu	62.1	476 (9.8)	7.7	476.7	8.2	7.7
Andhra Pradesh	75.7	299 (6.1)	3.9	461.1	7.9	6.1
Union Territory of Chandigarh	0.9	364 (7.5)	404.4¶	336.2	5.8	373.6¶
Kerala	31.8	183 (3.8)	5.8	177.5	3.0	5.6
Punjab	24.3	105 (2.2)	4.3	123.4	2.1	5.1
Gujarat	50.6	74 (1.5)	1.5	68.4	1.2	1.4
Madhya Pradesh	60.4	71 (1.5)	1.2	61.9	1.1	1.0
Union Territory of Pondicherry	1.0	66 (1.4)	66.0	53.7	0.9	53.7
Haryana	21.1	95 (1.9)	4.5	44.6	0.8	2.1
Orissa	36.7	41 (0.8)	1.1	43.2	0.7	1.2
Rajasthan	56.5	62 (1.3)	1.1	40.8	0.7	0.7
Jammu And Kashmir	10.1	20 (0.4)	2.0	28.6	0.5	2.8
Assam	26.6	18 (0.4)	0.7	26.0	0.4	1.0
Uttaranchal	8.5	21 (0.4)	2.5	25.7	0.4	3.0
Meghalaya	2.3	9 (0.2)	3.9	13.7	0.2	6.0
Himachal Pradesh	6.1	12 (0.2)	2.0	13.1	0.2	2.1
Andaman & Nicobar Islands	0.4	6 (0.1)	15.0	9.9	0.2	24.8
Goa	1.3	6 (0.1)	4.6	8.6	0.1	6.6
Bihar	82.9	5 (0.1)	0.1	5.5	0.1	0.1
Jharkhand	26.9	5 (0.1)	0.2	3.4	0.1	0.1
Sikkim	0.5	3 (0.1)	6.0	1.6	0.0	3.2
Manipur	2.4	4 (0.1)	1.7	1.5	0.0	0.6
Chhattisgarh	20.8	3 (0.1)	0.1	1.3	0.0	0.1
Arunachal Pradesh	1.1	2 (0.0)	1.8	0.9	0.0	0.8
Tripura	3.2	2 (0.0)	0.6	0.3	0.0	0.1
Top fifteen cities (State / Union Territory)*						
Delhi (National Capital Territory of Delhi)	13.8	1014 (20.8)	73.5	1216.0	20.8	88.1
Bangalore (Karnataka)	8.4	258 (5.3)	30.7	598.2	10.2	71.2
Mumbai (Maharashtra)	11.9	393 (8.1)	33.0	499.4	8.5	42.0
Kolkata (West Bengal)	4.6	299 (6.1)	65.0	463.6	7.9	100.8
Hyderabad (Andhra Pradesh)	3.7	233 (4.8)	63.0	404.2	6.9	109.2
Chandigarh (Union Territory of Chandigarh)	0.9	364 (7.5)	404.4¶	336.2	5.8	373.6¶
Lucknow (Uttar Pradesh)	3.7	272 (5.6)	73.5	332.1	5.7	89.8
Chennai (Tamil Nadu)	4.2	246 (5.0)	58.6	267.6	4.6	63.7
Pune (Maharashtra)	7.2	108 (2.2)	15.0	163.0	2.8	22.6
Varanasi (Uttar Pradesh)	3.1	87 (1.8)	28.1	138.8	2.4	44.8
Thiruvananthapuram (Kerala)	3.2	121 (2.5)	37.8	135.7	2.3	42.4
Mysore (Karnataka)	2.6	74 (1.5)	28.5	96.2	1.6	37.0
Vellore (Tamil Nadu)	3.5	84 (1.7)	24.0	95.6	1.6	27.3
Pondicherry (Union Territory of Pondicherry)	0.7	66 (1.4)	94.3	53.7	0.9	76.7
Visakhapatnam (Andhra Pradesh)	2.2	34 (0.7)	15.5	38.5	0.7	17.5

*Listed in descending order of total impact factor of health papers; the states / union territories of Mizoram, Nagaland, Dadra & Nagar Haveli, Daman & Diu, and Lakshadweep had no publications in PubMed in 2002

**†**Population for 2001 from the Census of India [14]

**‡**Percent of the total 4876 health research papers from India in 2002

**§**Percent of the total impact factor of 5842.055 for all 4876 health research papers from India

¶This high per capita output is likely related to the small population of Chandigarh and the high concentration of academic institutions

Search of websites of major academic institutions in India, international agencies, and publishing houses revealed that substantial original public health research output that was accessible in the public domain was not readily available from these sources. Among the major academic institutions in India involved with public health research, only one was found to have a few reports on health research accessible on its website [9] and another had some health research abstracts on its website [10]. The international agencies had some reports on their websites on India-related health research that were mostly authored by non-Indian authors.

In the April-June quarter of 2002, 1905 health papers published from Australia were located on PubMed, of which 722 (37.9%) were in basic sciences, 954 (50.1%) in clinical sciences, and 229 (12%) in public health. Taking into account the population and total gross domestic product (GDP) adjusted for purchasing power parity (PPP) of Australia and India [1], the quality-adjusted health research output and public health research output were 19.6 and 31 times higher from Australia than India, respectively, per unit GDP adjusted for PPP (Table 6).

**Table 6:** Comparison of health research output from India and Australia in 2002

	**India**			**Australia**			**Australia-India ratio**	
	
	**Total**	Per million population*	**Per billion GDP-PPP†**	**Total‡**	Per million population*	**Per billion GDP-PPP†**	**Per million population**	**Per billion GDP-PPP**
No. of health papers	4876	4.72	1.66	7620	392.78	15.49	83.2	9.3
Impact factor for health papers	5842	5.65	1.99	19231	991.27	39.10	175.3	19.6
No. of basic science papers	2358	2.28	0.80	2888	148.87	5.87	65.2	7.3
Impact factor for basic science papers	3944	3.82	1.35	10598	546.31	21.55	143.1	16.0
No. of clinical science papers	2296	2.22	0.78	3816	196.70	7.76	88.5	9.9
Impact factor for clinical papers	1698	1.64	0.58	7624	393.01	15.50	239.1	26.7
No. of public health papers	216	0.21	0.07	916	47.22	1.86	225.9	25.3
Impact factor for public health papers	193	0.19	0.07	1008	51.95	2.05	277.6	31.0

*Based on 1033.4 million population for India and 19.4 million for Australia in 2001 [1]

**†**Based on the gross domestic product adjusted for purchasing power parity (GDP-PPP) of US$ 2930 billion for India and US$ 491.8 billion for Australia in 2001 [1]

**‡**Based on multiplying the number of papers and their total impact factor for the April-June 2002 quarter by four to obtain the estimate for the year 2002

The total of the number and impact factor for basic, clinical and public health papers does not add up to that for the health papers, as 6 "other" papers (with total impact factor 6.012) that could not be classified as basic, clinical or public health (Table 1) are not included in this table

## Discussion

The data presented in this paper suggest that the health research output from India is not commensurate with the magnitude and distribution of disease burden. The research output in public health is particularly meagre, which is a major concern as public health sciences are a necessary tool to facilitate improvement in population health. Within this low research output, several diseases/conditions contributing substantially to the disease burden and several major areas of public health importance have relatively less representation. Without dynamic, relevant, good quality and adequate original research in the various aspects of public health it is difficult to imagine how the sub-optimal health status of the Indian population would improve on rhetoric or theoretical concepts alone [14,15].

In this paper we used impact factors for journals as a measure of the quality of papers published in those journals. Although impact factors are not without their limitations, they still offer a tangible, and perhaps the best available, option to compare the quality of publications in journals [6].

We explored several sources where information about health research output from India could be available in the public domain, as the utilisation of research findings is facilitated most if they are readily accessible in the public domain. However, we did not find any source that would add substantially to the information available in the PubMed database. Indeed, there are more Indian health journals than are included in PubMed, but their quality in general is not as high as those included in PubMed with none of them having an impact factor above zero. Non-inclusion in our analysis of the papers published in these journals, therefore, did not bias our assessment of quality-adjusted research output based on impact factors. The relative low quality and impact factor of a large proportion of Indian journals has been discussed previously [16,17]. PubMed lists affiliation of the first author only, and therefore, the analysis presented in this paper includes only those publications in which the first author had Indian affiliation. There would be other publications with non-Indians as first author and Indians as co-author(s), which we estimate to be a very small fraction of those with Indians as first author. In the general context, the PubMed/MEDLINE database has been used previously to assess the health research output from several countries [18-25].

We used the disease burden in India as estimated by the Global Burden of Disease Study [2]. Although the limitations of this Study have been debated previously in the literature, we could not find a better alternative for use for our study, as these were the most comprehensive estimates available for India. In any case, these estimates can be taken only as indicative, and therefore, we highlight only gross deviations of health research output from these trends.

There has been a previous attempt to assess the health research output from India using the Science Citation Index of 1981–85 and relating the number of papers published in journals of various medical/health specialities with the perceived areas of major disease burden [26]. However, review of all published abstracts to classify each paper in various categories, the approach used by us, has not been used previously to assess health research output from India to our knowledge. Systematic tracking of health research output, and its relation to the estimated trends in disease burden, are necessary for guiding further appropriate development of health research in India. In addition to the overview of research needs identified in this paper, more in-depth assessment of research needs for major diseases/conditions would also be necessary, as was reported recently for the evidence base needed to control HIV/AIDS in India [27].

Since public health sciences seem to be the weakest link in improving health in India currently, it is imperative that a strategic framework for developing original public health research in India be evolved. To do so, the *demand*, *supply *and *environment *issues would have to be addressed:

• **Demand. **Among the multitude of factors that influence the demand for relevant public health research, the role of policy makers and senior health academics is of particular importance. This is seriously sub-optimal in India at present. Political compulsions push many policy makers into short-term gains instead of investments in comprehensive research for long-term benefits. Although there has recently been an increasing trend in India towards commissioned research by government and international agencies in some aspects of public health, this by itself is not enough to boost comprehensive public health research in India, and the reports of such studies are many times not available in the public domain which reduces the chance of their widespread utilisation. Many senior health academics in India continue to disregard public health research as a less-respectful cousin of basic and clinical research. Systematic efforts are needed to demonstrate to these groups the linkages between all aspects of health research (basic, clinical and public health), and the linkages between public health research and improvements in population health, in order to boost the demand for relevant and good-quality public health research in India.

• **Supply. **Enhancing the output of public health research will require effort on various fronts. Establishing schools of public health and other institutions to train quality scientists in public health is a priority, as India has a surprisingly few number of institutions that can provide proper training in public health research. Another area that needs quick attention is to make public health exposure in medical and paramedical colleges more practical to encourage hands-on investigative thinking, as currently it is so theoretical that it rarely inspires enthusiasm in young professionals towards public health research. Setting higher standards for the research dissertations currently required for post-graduate degrees in preventive and social medicine would also encourage better quality and practically relevant public health research. It is also necessary to systematically develop performance-based opportunities to public health research scholars for career enhancement. Another element that would help develop public health research capacity in India is evolving mechanisms to encourage contribution to this effort by the many Indian public health researchers living abroad.

• **Environment. **A conducive environment is necessary for the demand and supply of public health research to function optimally. Efforts are needed to develop this by attempting to develop broad-based coalitions, that include health care providers, civil society and non-governmental sector, for-profit private sector and industry, and national and international agencies providing financial support, which would understand and support the need for vibrant public health research as a vital element of societal development. This is a necessary element that has so far received scant attention, which must be addressed if sustainable development of public health research to improve population health is to become possible in India. An environment of good-quality and comprehensive public health research in India would also infuse the much-needed originality in teaching public health sciences and their practical application to the local context.

Evolving such frameworks would require building up a critical momentum for this effort through perseverance and wisdom. One such opportunity is provided by the recent initiative of the Indian Ministry of Health and Family Welfare to develop more effective institutes of public health in India, with relevant public health research and its utilisation an important key to improving population health [28].

The recent attention towards revitalising the academic aspects of health care / medicine through evidence [29] and evidence-based global health [30] is particularly relevant for developing nations. Evolving a strong, dynamic and locally-relevant evidence base is even more important for developing nations as this is likely to yield relatively higher returns by contributing to improvements in the health, lives and economy of a larger proportion of the world's population. For this to happen, theoretical concepts alone would obviously be not enough. The practical solutions for this effort would have to be developed wisely. The data and its interpretation presented in this paper are, we hope, an example of how the deficiencies in the evidence-base needed for adequate health care in developing nations can be understood objectively in order to plan its strengthening.

## Conclusions

• Publications from India in PubMed were 11 times less in public health than those in basic sciences and in clinical sciences in 2002.

• Injuries, cardiovascular diseases, respiratory infections, diarrhoeal diseases, perinatal conditions, childhood cluster diseases, unipolar major depression, and HIV/AIDS had substantially less proportion of quality-adjusted original research output in India as compared with their contribution to the disease burden.

• India produced 20 times less quality-adjusted health research output than Australia per unit gross domestic product adjusted for purchasing power parity, and this ratio for public health research output was even higher at 31 times.

• Good-quality public health research output from India is grossly inadequate, and strategic planning to improve it is necessary if substantial enhancement of population health were to be made possible.

## Competing interests

The author(s) declare that they have no competing interests.

## Authors' contributions

LD conceived the idea of this study, guided the design, data collection and analysis, and wrote the initial draft of this paper. YSS contributed to the design, data collection and analysis. MNJ contributed to data collection and analysis. VSUB contributed to data management and analysis. RD contributed to the idea of this study, design and data analysis. All authors read and approved the final manuscript.

## Pre-publication history

The pre-publication history for this paper can be accessed here:


